# Building Environmental and Sociological Predictive Intelligence to Understand the Seasonal Threat of SARS-CoV-2 in Human Populations

**DOI:** 10.4269/ajtmh.23-0077

**Published:** 2024-02-06

**Authors:** Moiz Usmani, Kyle D. Brumfield, Bailey Magers, Aijia Zhou, Chamteut Oh, Yuqing Mao, William Brown, Arthur Schmidt, Chang-Yu Wu, Joanna L. Shisler, Thanh H. Nguyen, Anwar Huq, Rita Colwell, Antarpreet Jutla

**Affiliations:** ^1^ GeoHealth and Hydrology Laboratory, Department of Environmental Engineering Sciences, University of Florida, Gainesville, Florida;; ^2^ Maryland Pathogen Research Institute, University of Maryland, College Park, Maryland;; ^3^ University of Maryland Institute for Advanced Computer Studies, University of Maryland, College Park, Maryland;; ^4^ Department of Civil and Environmental Engineering, University of Illinois at Urbana–Champaign, Urbana, Illinois;; ^5^ Department of Environmental Engineering Sciences, University of Florida, Gainesville, Florida;; ^6^ Department of Pathobiology, University of Illinois at Urbana–Champaign, Urbana, Illinois;; ^7^ Department of Chemical, Environmental and Materials Engineering, University of Miami, Florida;; ^8^ Department of Microbiology, University of Illinois at Urbana-Champaign, Urbana, Illinois

## Abstract

Current modeling practices for environmental and sociological modulated infectious diseases remain inadequate to forecast the risk of outbreak(s) in human populations, partly due to a lack of integration of disciplinary knowledge, limited availability of disease surveillance datasets, and overreliance on compartmental epidemiological modeling methods. Harvesting data knowledge from virus transmission (aerosols) and detection (wastewater) of SARS-CoV-2, a heuristic score-based environmental predictive intelligence system was developed that calculates the risk of COVID-19 in the human population. Seasonal validation of the algorithm was uniquely associated with wastewater surveillance of the virus, providing a lead time of 7–14 days before a county-level outbreak. Using county-scale disease prevalence data from the United States, the algorithm could predict COVID-19 risk with an overall accuracy ranging between 81% and 98%. Similarly, using wastewater surveillance data from Illinois and Maryland, the SARS-CoV-2 detection rate was greater than 80% for 75% of the locations during the same time the risk was predicted to be high. Results suggest the importance of a holistic approach across disciplinary boundaries that can potentially allow anticipatory decision-making policies of saving lives and maximizing the use of available capacity and resources.

## INTRODUCTION

Published reports indicate that aerosols are now considered a critical route of transmission for respiratory viruses.^1^^–^^5^ Viable SARS-CoV-2 particles have been collected from the air at a distance of up to 4 m from infected patients.^3^ Several studies suggest SARS-CoV-2 becomes airborne in the built infrastructure or outdoors given favorable ambient environmental conditions.^3^^,^^6^^,^^7^ Large droplets containing SARS-CoV-2 tend to settle on the ground within 1–2 m,^8^ whereas smaller droplet nuclei remain airborne (including viruses attached to solid or liquid particulates), constituting a major transmission route for humans under prevailing ambient weather and climatic conditions. Although an infectious dose can vary and be influenced by a range of host characteristics (age, sex, comorbidities, smoking, etc.), as well as genetic mutation of the virus itself, respiratory viruses infect human hosts, proliferate, and spread from the hosts via air. Clinical diagnosis of coronavirus disease (COVID-19) infection is by detecting SARS-CoV-2 RNA in nasopharyngeal swabs. However, SARS-CoV-2 RNA is also present in feces of infected individuals.^9^^,^^10^ Moreover, SARS-CoV-2 RNA is detectable in fecal samples several days before development of symptoms in humans. Viral shedding in feces has been reported to persist in clinical samples that tested negative.^10^ Hence, wastewater surveillance (WS, also referenced as wastewater monitoring or wastewater-based epidemiology) of wastewater treatment systems is now practiced as a complementary tool to clinical testing.^11^^–^^13^ A few studies employing wastewater sampling have reported community transmission of SARS-CoV-2 before onset of COVID-19 cases.^11^^,^^14^^,^^15^ That is, SARS-CoV-2 variants in a community have been shown to be present in wastewater before clinical cases were reported,^13^^,^^16^ suggesting longitudinal wastewater analysis can be used to detect viral shedding by infected (symptomatic and asymptomatic) individuals, hence has potential to identify outbreaks earlier than clinical case reports.

It was reported that SARS-CoV-2 can aerosolize in cold, dry environments, notably at low dew point temperatures when the difference between ambient air and dew point temperatures is high.^17^ When ambient air temperatures are either greater than 24 or less than 17°C, transmission of SARS-CoV-2 increases in the human population and is associated with changes in human behavior and modes of interaction in the built environment. However, within an ambient air temperature range of 17–24°C, transmission lessens, reducing the number of human infections. In summary, the number of reported cases within a population will depend on the local weather and climatic variables, all of which provide background for the rate of newly reported cases.

After the COVID-19 pandemic began, several mathematical modeling strategies were offered, mainly to understand transmission of COVID-19 in human populations. Typical among these were simulation of COVID-19 infections on college campuses,^18^^,^^19^ assessment of potential mortality in the United States,^20^ and compartmental epidemiological models with various modes of population heterogeneity^21^^,^^22^ and fluid dynamics-based methodologies for respiratory droplets and aerosols.^23^ Although each modeling strategy has utility, prediction of COVID-19 risk for human populations is limited. Most of the COVID-19 models are variants of compartmental epidemiological models (Susceptible–Infected–Recovered architecture) and therefore lack predictive intelligence because they require 1) initial values to initiate model simulation and 2) hypothetical excitation values of reproductive numbers, which are a function and thus require incidence or prevalence time series of COVID-19. These models and other regressive techniques (machine learning,^24^^,^^25^ regression models^26^^,^^27^) lack parsimony and generalization ability, implying the simulations remain native to geographical locations.

SARS-CoV-2 provides a unique opportunity because it is known that 1) the virus can be detected in wastewater several days before an outbreak occurs in a community^28^^,^^29^ and 2) a respiratory route of infection occurs via virus-laden aerosolized particles.^30^^,^^31^ The criticality of the relationship between transmission (air) and detection (wastewater) is key to developing predictive intelligence^32^^–^^34^ (broadly defined as collective knowledge harvested from multiple data sources for a mathematical risk and decision-making assessment system) for forecasting risk of COVID-19. The important question—and the goal of this study—is whether reliable predictive intelligence can be developed using collective knowledge from the air–water nexus to forecast seasonal threat of COVID-19 in human populations. Emerging evidence indicates COVID-19 will be seasonal^17^; however, seasonality is viewed as a function of geographic space and time, implying specific regions of the world will be impacted during a particular period of time more so than at other times of the year. This poses a critical problem in determining when and where to intervene strategically—that is, intervention will be more effective if detecting an outbreak of COVID-19 is reliable through surveillance of wastewater for SARS-CoV-2 RNA.

It is important to distinguish categorically between prediction and simulation modeling systems. Prediction is likely to provide estimates for the true unknown (in the time domain), while simulation systems are likely to be scenario generation algorithms. Current epidemiological models greatly enhance simulation capability but lack risk predictive capacity. A true predictive modeling system will provide risk of COVID-19 in a given space and time over a particular region, the objective of this study. Hence, we developed a unique spatial and temporal scale-dependent and socioenvironmental driven predictive scoring system to answer these questions. The model was validated using county scale data for the continental United States and finer community-level scale using WS data. The predictive system presented in this study encapsulates the climate, weather, and sociological attributes associated with the virus and captures the inherent stochastic behavior of viral transmission, allowing replication of outputs in different regions and providing parsimonious versatility.

## MATERIALS AND METHODS

On the basis of our previous hypothesis,^17^ five variables were used to develop the predictive system: ambient air temperature, dew point temperature, population density, ethnicity, and household income. Environmental variables (dew point temperature and ambient air temperature) were obtained on a daily scale from Oregon State University’s Parameter-Elevation Regressions on Independent Slopes Model (PRISM) climate group products at a resolution of 4 km × 4 km (PRISM Climate Group, Oregon State University, http://prism.oregonstate.edu). Socioeconomic (https://www.census.gov/data/datasets/time-series/demo/popest/2010s-counties-total.html#par_textimage_70769902), ethnicity, and household income data sets were retrieved from the U.S. Census Bureau. The population density data were obtained from Oak Ridge National Laboratory’s LandScan population data at 1 km × 1 km resolution. The reported daily number of COVID-19 cases at the county level in the United States were downloaded from an open-source upstream repository (https://github.com/datasets/covid-19) maintained by Johns Hopkins University Center for Systems Science and Engineering (CSSE). On a finer scale, the daily COVID-19 cases were provided by the Champaign-Urbana Public Health District and the Maryland Department of Health for Illinois and Maryland, respectively.

The core of the methodology for the algorithm was developed using a heuristic score-based mathematical set of rules by modifying our previously developed cholera forecasting system.^35^^,^^36^ The algorithm is based on weighted raster architecture where appropriate weights are provided to each variable of interest and a composite mean is computed that translates to a risk score.

### Identification of environmental variables.

Various environmental variables have been identified to impact the transmission of COVID-19 significantly. It was triggered in a cold region during the winter months and advanced into other colder regions during winter and spring. Once the summer started, disease transmission moved towards the warmer/hot regions, decreasing the number of reported cases in the colder regions. This decline in cases coincided with the comfortable ambient air temperature range of 17–24°C in the colder regions. We identified a decrease in cases if a region experiences ambient air temperature between 17 and 24°C; therefore, regions on either side of this range experience more cases. These variabilities in the reported cases, according to the temperatures, are indicative of human behavior beyond the comfortable temperature range. We further determined that a region that experienced a combination of negative dew point temperature and an excess of 5°C difference between ambient air temperature and dew point temperature has reported more infected cases than other colder regions during winter and spring. The positive association of cold and dry weather with the number of infected cases suggests rapid aerosolization of SARS-CoV-2.

Along with environmental variables, various socioeconomic variables have been identified to significantly associate with the transmission of the disease in society. The population density was identified as the most crucial variable of infectious illnesses in positively determining the infected cases in a region that directly correlates disease transmission.^37^^–^^39^ However, because it is hard to estimate an average spatial threshold of population density for an outbreak of a specific infectious disease, we used ordinal logistic regression to determine the association of population density over the COVID-19 reported cases in the United States at a county scale.^40^ Apart from population density, the other socioeconomic factors that we identified as significant are income and ethnicity.^41^^–^^43^ Historically, the spread of infectious diseases has been associated with household income. Low-income households have exhibited a strong positive association with COVID-19 cases. For this study, we defined household income less than $35,000 USD as a low income.^44^^,^^45^ The Centers for Disease Control and Prevention (CDC) has identified ethnicity as an indicator of the community’s spread of COVID-19 due to its strong association with healthcare access and occupational exposure to the virus due to socioeconomic status. According to the CDC report, the African American and Hispanic populations are at 2.6 and 2.8 times higher risk of infection, respectively, compared with the White and Non-Hispanic populations.^46^ Similarly, the African American and Hispanic populations are at 4.7 and 4.6 times higher risk of getting hospitalized, respectively.

Incorporating ambient air temperature, dew point temperature, population density, ethnicity, and household income, we developed a model to predict the spatiotemporal COVID-19 risk.

### Quantification of variables.

We analyzed the previous 2 weeks (in context to risk score prediction date) of ambient air temperature for each pixel (1 km × 1 km) by categorizing it based on the tolerable temperature (17–24°C).^17^ Any temperature within the comfortable range was considered to have the least impact on the transmission of the virus in the human population. Deviation lower than 17°C or higher than 24°C was computed by the square root for homogeneity of variance in the variable. The deviation from 17 to 24°C was capped at 16°C, such that any value greater than 16°C would be considered as 16°C. Therefore, the square root of deviation varied between 0 and 4 as real numbers. The difference between ambient air temperature and dew point temperature was used to quantify the cold and dry conditions for the air. Here, cold regions were defined as regions that experience below freezing temperatures (for one or more days) for 3 or more months. The difference between ambient air temperature and dew point temperature was determined on a daily scale for 2 weeks before predicting the risk score. Lastly, these daily differences were masked with the negative dew point temperature, with negative dew point temperature as 1 and positive as a value of 0. Therefore, it represented cold and dry climatic conditions over the region experiencing negative dew point temperature. Similarly, it was classified on a scale of 0 (minimum) to 4 (maximum). Zero risk score (0) if dew point temperature was positive, low-risk score (1) if the variable was between 0 and 3.5, medium risk score (2) if the variable was between 3.5 and 7, high risk score (3) if the variable was between 7 and 11.5, and very high-risk score (4) if it was greater than 11.5. The next step was to determine the weight of these environmental parameters for the predictive model. Both environmental parameters are summed up with weights varying between 0.5 and 0.9 for the first parameter and 0.1–0.5 for the second parameter in the combination shown in Supplemental Figure 1. The environmental variable of the model with weights of 0.8 and 0.2 of the first and second parameters, respectively, exhibited the best results in terms of the statistical measures. Thus, the environmental variable was determined by summing up and normalizing both environmental parameters in a ratio of 4:1. The environmental risk computed using these variables for the first 3 weeks of April 2020 is shown in Supplemental Figure 1.

In this study, the population density was considered the most crucial socioeconomical variable, logarithmically quantified on a scale of 0 (minimum) to 4 (maximum). The second and third variables, ethnicity and household incomes, are taken in ratios at a county level. We formulated the socioeconomical variable with a heuristic approach and provided 0.8, 0.1, and 0.1 weights for population density, ethnicity, and income. All three variables were on a scale of 0–1, so the final socioeconomical variable follows the same scale. The socioeconomical risk of COVID-19 is shown in Supplemental Figure 2.

The overall disease transmission risk was calculated as the product of environmental and socioeconomical risks. Therefore, only social factors play a role in determining the risk within the comfortable temperature range, but climatic variables influence the transmission rate beyond that range.

### Wastewater surveillance.

#### Site description and sample collection.

Wastewater surveillance was carried out by monitoring components of sewer collection systems in Illinois and Maryland (locations are provided in Supplemental Figure 2). The total daily number of reported COVID-19 cases in Illinois and Maryland residences within the zip codes where the study was conducted were retrieved from the Champaign–Urbana Public Health District and the Maryland Department of Health, respectively. Composite samples were collected weekly from manholes downstream of the sewer collection system in Illinois and Maryland between September and December 2021, using an automated composite sampling unit (Teledyne ISCO, Lincoln, NE) with ice surrounding the collection jar. During each sampling event in Illinois, samples (∼60 mL) were collected at 4-hour intervals over 4 days, totaling 24 individual sampling attempts and a composite sample volume of 1–2 L. Similarly, during each sampling event in Maryland, samples (60 mL) were collected at 15-minute intervals for 24 hours, totaling 96 individual sampling attempts and a composite sample volume of 5.76 L.

#### Illinois sample processing.

Illinois samples were processed according to methods described previously.^47^ The sewage sludge was concentrated by centrifugation for pretreatment. Briefly, 20 mL of 2.5 M MgCl_2_ was added to each sewage sample for flocculation at the sampling site. After arriving at the laboratory, the sewage samples were stored at 4°C pending pretreatment. All samples were pretreated within 24 hours after the samples’ arrival. The actual total volumes of the sewage samples ranged between 80 and 2,935 mL. However, most of the sample volumes were between 1,000 and 2,000 mL. Before centrifugation, the supernatant of the sewage sample was removed. Approximately 300 mL of the liquid with sludge was retained in the sample bag. The removed supernatant was temporarily stored in 1,000 mL clean glass bottles with graduation lines to calculate the actual total volume of the sewage sample and measure the sample pH. Sample pH was measured using Hydrion 6.0–8.0 pH paper (Micro Essential Laboratory, Brooklyn, NY). The remaining liquid with sludge in the sampling bag was then transferred to 50-mL sterilized polypropylene centrifuge tubes, 35 mL each for centrifugation. Next, 350 µL of water-dissolved Bovilis Coronavirus Vaccine (Merck Animal Health, Rahway, NJ) was spiked in each centrifuge tube to calculate the recovery rate of SARS-CoV-2 RNA. The samples were centrifuged at 15°C at a speed of 10,000 × *g* for 30 min. After centrifugation, the supernatant was discarded. Approximately 0.1 mL of the pellet was taken for RNA extraction. If any liquid remained in the sample bag after splitting the liquid for centrifugation, the remaining liquid would be stored in 50-mL centrifuge tubes to calculate the actual total sample volume and do other analyses. Three parts of the liquid volumes were combined to determine the total sample volume calculation, including the supernatant temporarily stored in 1,000-mL glass bottles, the liquid with sludge for centrifugation, and the remaining liquid in the sample bag.

Viral RNA was extracted from the 0.1-mL pellet using QIAmp Viral RNA Mini Kit (Qiagen, Düsseldorf, Germany) following the manufacturer’s protocol. The number of viral genomes was quantified by Taqman-based reverse transcriptase qualitative polymerase chain reaction (RT-qPCR). The Taqman-based RT-qPCR was initiated by mixing 5 μL of viral genome with 5 μL of Taqman Fast Virus 1-step Master Mix (4444432, Applied Biosystems, Waltham, MA), 1.5 μL of primers/probe mixture for N1 gene (2019-nCoV RUO kit, Integrated DNA Technologies, Coralville, IA), and 8.5 μL of water. The 20 μL of RT-qPCR cocktail was placed in the 96-well plates (4306737, Applied Biosystems), and the RT-qPCR was run using a qPCR system (Quant Studio 3, Thermo Fisher Scientific, Waltham, MA) with the following thermal cycle: 5 minutes at 50°C, 20 seconds at 95°C, followed by 45 cycles of 3 seconds at 95°C and 30 seconds at 55°C. Standard curves were obtained for every RT-qPCR analysis with 10-fold serial dilutions of synthetic RNA controls (102024, TWIST Bioscience, San Francisco, CA), and PCR efficiencies were higher than 85% (R2 > 0.99). The information for RT-qPCR primers is shown in Supplemental Figure 3.

#### Maryland sample processing.

Maryland samples were processed according to methods described previously.^13^ Briefly, samples were homogenized manually, and an aliquot (110 mL) was pasteurized in a water bath at 60°C for 30 minutes, transported to the laboratory on ice, and processed the same day. To remove larger debris, 45 mL was transferred to a sterile polypropylene 50-mL conical tube and centrifuged at 7,500 RCF for 10 minutes at 4°C. The resulting supernatant was transferred to a clean conical tube and concentrated using InnovaPrep Concentrating Pipette Select with Ultrafiltration PS Hollow Fiber pipette tips (InnovaPrep, Drexel, MO), following manufacturer’s recommendations (“Wastewater Application Note, Revision B”). InnovaPrep Wet Foam Elution was stored in DNA/RNA shield (Zymo Research, Irvine, CA), following manufacturer’s specifications, at –80°C until nucleic acid was prepared (< 48 hours).

#### RNA purification.

To serve as an internal control (IC) for the RT-qPCR assay, 25 µL of MS2 Bacteriophage, target titer of 1.0’ 10^3^ pfu/mL (ZeptoMetrix, Buffalo, NY), was added to 115 µL InnovaPrep Wet Foam Elution. Total RNA was prepared from wastewater concentrates containing MS2 Bacteriophage, employing the QIAamp Viral RNA Mini Kit (Qiagen, Germantown, MD), following the manufacturer’s instructions for use on the automated QIAcube Connect platform. RNA extracts were stored in LoBind microcentrifuge tubes (Eppendorf, Hamburg, Germany) at –80°C before RT-qPCR amplification (< 48 hours).

#### Reference RNA materials.

The SARS-CoV-2 Research Grate Test Material (RGTM 10169; National Institute of Standards and Technology, Gaithersburg, MD), consisting of two synthetic RNA fragments from the SARS-CoV-2 genome (including SARS-CoV-2 sequences 25949–29698 and 12409–15962 of isolate USA-WA1/2020) in a background of 5 ng/µL human Jurkat RNA, was diluted over six serial log dilutions with nuclease-free water for use as calibration standards. RNA template of SARS-CoV-2 Nucleocapsid Phosphoprotein (N protein) encapsulated in the MS2 bacteriophage construct (PerkinElmer, Waltham, MA) was prepared following manufacturer’s instructions for use as a positive control (PC). Single-use aliquots of reference RNA were stored in LoBind microcentrifuge tubes (Eppendorf, Hamburg, Germany) at –80°C.

#### RT-qPCR amplification.

The New Coronavirus Nucleic Acid Detection Kit v.7.0 (PerkinElmer, Waltham, MA) was used on the QuantStudio^TM^ 3 System (Thermo Fisher, Waltham, MA), for multiplexed detection of SARS-CoV-2 N protein and IC, following manufacturer’s specifications. Following each run, log fluorescence thresholds were set manually, and melt curve analysis was done to identify spurious amplicons that could confound data interpretation (no spurious amplicons detected). Quantification cycle (Cq) values were exported to Excel (Microsoft, Redmond, WA) for further analysis.

#### Quality control.

Method extraction controls (PC, NC, IC) were prepared using the New Coronavirus Nucleic Acid Detection Kit (PerkinElmer) to monitor sample processing. Synthetic SARS-CoV-2 N protein RNA template encapsulated in MS2 bacteriophage (PerkinElmer) was included as PC. For IC, bacteriophage MS2 was used to monitor the process from nucleic acid extraction to fluorescence detection for amplification inhibition. Failure to detect PC resulted in an invalid run for all samples. Samples negative for IC, suggestive of amplification inhibition, or positive for NC, suggestive of potential contamination, were discarded from further analysis.

## Statistical analysEs

COVID-19 is a relatively new disease, and therefore a reliable long-term time series is not available where traditional statistical measures,^48^ such as coefficient of determination, root mean square, bias, or Nash–Sutcliffe Efficiency can be computed. Hence, we used accuracy, sensitivity, specificity, and precision to validate the model.^48^ These statistical measures are defined as

$$\text{Accuracy}=\left({\text{t}}_{\text{p}}+{\text{t}}_{\text{n}}\right)/\left({\text{t}}_{\text{p}}+{\text{t}}_{\text{n}}+{\text{f}}_{\text{p}}+{\text{f}}_{\text{n}}\right)$$ [1]

$$\text{Sensitivity}={\text{t}}_{\text{p}}/\left({\text{t}}_{\text{p}}+{\text{f}}_{\text{n}}\right)$$ [2]

$$\text{Specificity}={\text{t}}_{\text{n}}/\left({\text{t}}_{\text{n}}+{\text{f}}_{\text{p}}\right)$$ [3]

$$\text{Precision}={\text{t}}_{\text{p}}/\left({\text{t}}_{\text{p}}+{\text{f}}_{\text{p}}\right)\text{,}$$ [4]

where t_p_ = true positive, representing number of locations where the threshold of the reported cases is achieved when the computed risk is higher than the stated risk; t_n_ = true negative, representing number of locations where the threshold of reported cases is not achieved when the computed risk is lower than the stated risk; f_p_ = false positive, which represents number of locations where the threshold of reported cases is not achieved when the computed risk is higher than the stated risk; f_n_ = false negative, which represents the number of locations where the threshold of the cases is achieved when the computed risk is lower than the specifically stated risk.

To compute these statistical metrics, we evaluated computed risk scores and corresponding reported cases at the county scale by varying risk score values from 0.2 to 1.0 at an incremental step of 0.01. The motivation for this approach was to determine an optimum predicted risk score to serve as threshold. At each step, the varying value was compared with the computed risk score of a county, after which that county was categorized. Once each county was categorized at a specific value, the statistical measures were determined. The same approach was used for the next incremented value. Statistical measures for each value provided the base to evaluate model performance at varying risk values to determine the risk score threshold.

**Figure 1.**
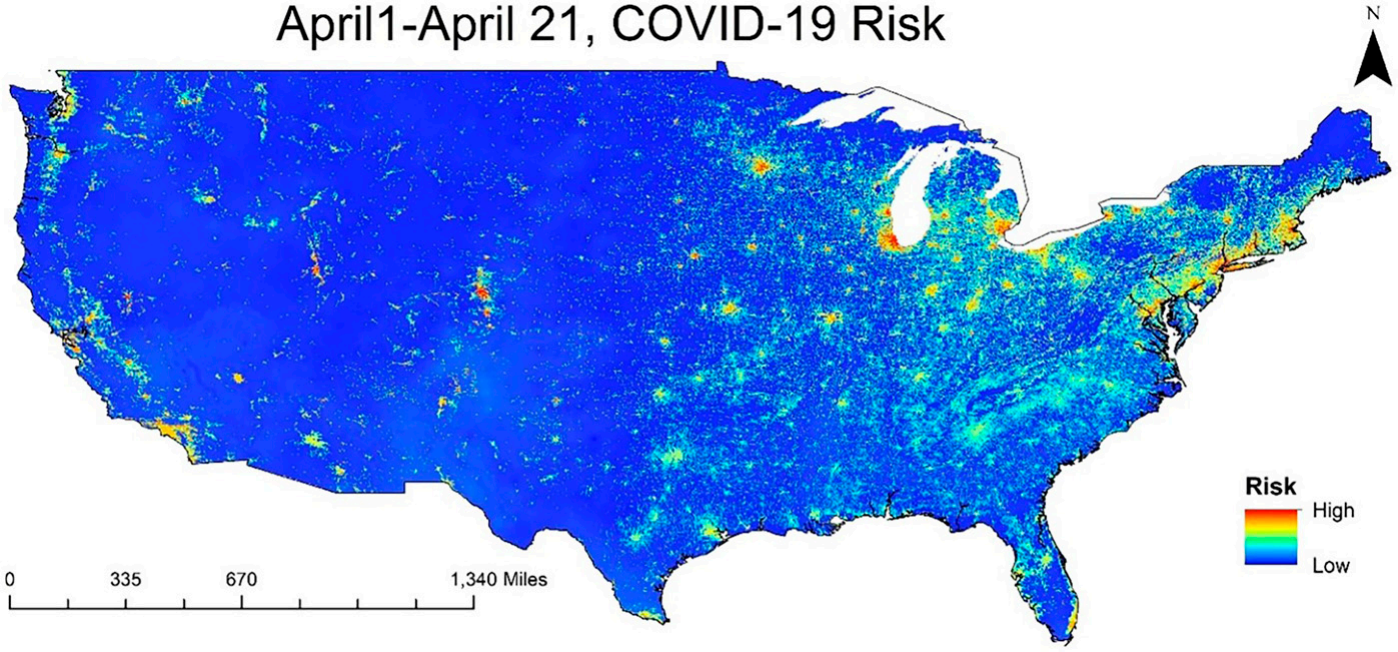
COVID-19 predicted risk map for April 1 to April 21 computed on March 31, 2020.

Detection of SARS-CoV-2 RNA in wastewater is shown as qualitative (presence/absence) data and is intended for model evaluation and not a direct comparison between sites. Site description, data collection, and processing steps are available in detail in supplementary section A. The prediction system was developed and subsequently validated for the continental United States, and risk scores were computed at a resolution of 1 km × 1 km (Figure 1). The model was validated using different time periods: March 1 through 21 (Spring 2021), June 1 through 21 (Summer 2020 and 2021), September 1 through 21 (Fall 2020 and 2021), and December 1 through 21 (Winter 2020), along with April 1 through 21 (2020). April 2020 represented conditions at the beginning of the outbreak in the United States. The predictive system output is a composite of static socioeconomic variables and time-varying dynamic environmental variables (Supplemental Section A). A known challenge of any disease risk prediction algorithm is validation, due to discrepancies in availability of spatial resolution of reported cases data and associated model outputs. Therefore, we validated the risk scores with the corresponding reported cases at the average county scale and pixel scale at two spatial resolutions. Choice of the pixel scale risk score is critical for designing intervention strategies by accurately identifying the location. The risk scores must be computed at the same spatial scale as disease-reported cases data; thus, the risk score at a county scale was computed using two methods. First, risk scores were computed with pixel average at the county scale (R_p_). Then the maximum risk score within the county was determined and stated as maximum risk (R_m_). Conceptual structure is provided in the flowchart shown in Supplemental Figure 3. A confusion matrix was created for three thresholds of the number of reported cases at the county scale: average (a_v_), that is, average plus one standard deviation (a_v1_), and average plus two standard deviations (a_v2_) at an incremental step of 0.01 of risk scores.

Model performance was evaluated using statistical metrics, derivative of the confusion matrix that further determines risk score thresholds for this disease. Statistical parameters used in this study were accuracy, precision, sensitivity, and specificity. A combination of these four statistical parameters served to evaluate model performance. Model performance, in terms of these statistical measures for the three thresholds at an incremental step of 0.01, is shown in Supplemental Table 4. For example, for risk value of 0.20, all four statistical metrics were determined and further calculated for all values between 0.2 and 1 at an increment of 0.01. Risk score threshold values were determined for each disease threshold, employing the optimum value of all statistical parameters and the optimum value by average value of the four statistical parameters without biasing individual parameters.

## RESULTS

Outbreak of a new disease in a human population poses an inherent predictive modeling challenge, namely, validation of a mathematical algorithm and its application, as well as translation of the modeled risk to new regions where disease prevalence data are limited or unavailable, coupled with emerging etiology of the disease. Our score-based predictive intelligence system aimed to overcome the two challenges, requiring unique interpretation while validating computed scores. The scores (risk scores) ranged from 0 to 1 and were calibrated based on a_v_, a_v1_, and a_v2_. Thus, identifying a risk between 0 and 1 was critical in determining the value above which a particular region would be expected to have an above average number of COVID-19 cases. This process is unique and overcomes translation of risk scores for other regions, as shown in Supplemental Figure 3. There are a few constraints on using county scale infection data: 1) it is not able to capture the distribution of reported cases within the county, 2) it also poses a challenge for the validation of finer scale model, and 3) it also poses a challenge to locate wastewater sampling sites. The risk scores are computed at the county scale with pixel average (R_p_) and maximum pixel value (R_m_) to overcome these constraints. The zip code scale data are used for wastewater sampling validation, which is at a much finer scale than the county.

### County scale calibration and validation.

**Figure 2.**
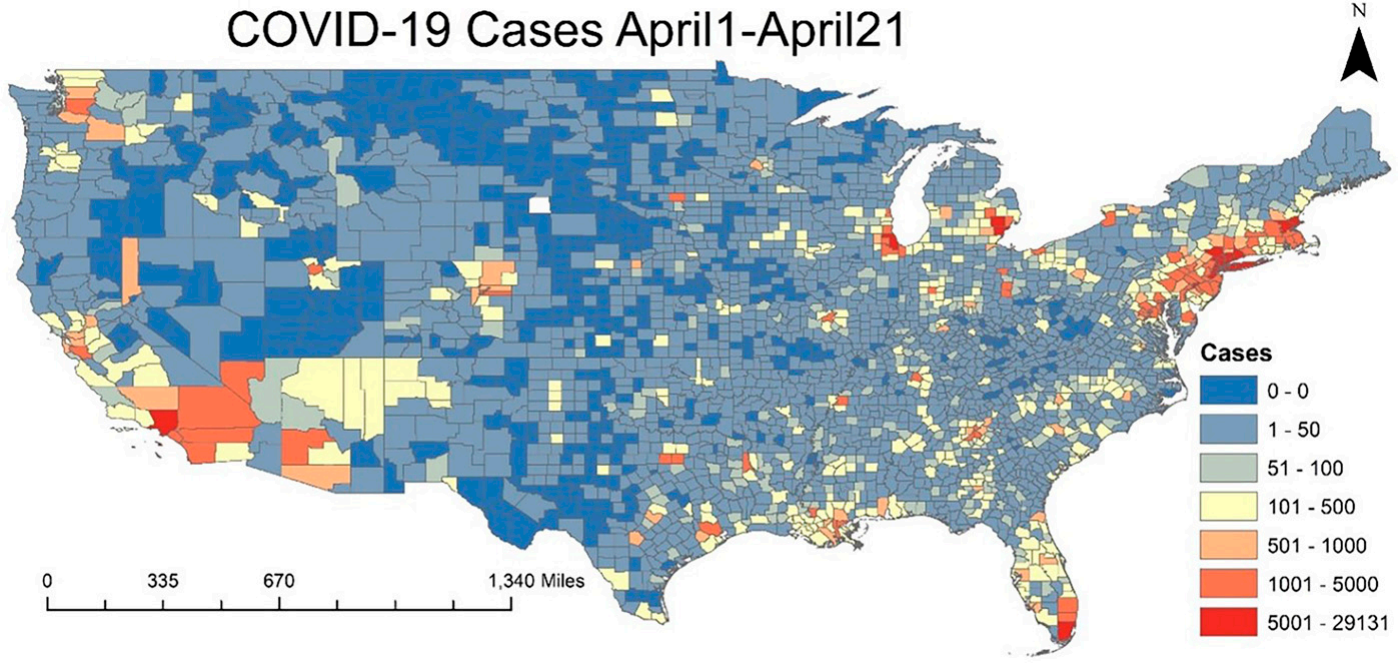
Reported cases at county scale between April 1 and April 21.

Calibration of the model was first on county scales. Risk scores (R_p_) for the first 21 days of April 2020 were calculated. Risk score threshold (or calibrated) values for a_v_ (201)_,_ a_v1_(1,558), and a_v2_(2,915) were 0.39, 0.50, and 0.55, with average statistical metrics of 76%, 75%, and 74%, respectively, as shown in Table 1. The model was used for six periods spanning all seasons in the continental United States. During winter (December), more cases were reported for the northern part of the country, whereas during summer (June) the northern region reported fewer cases. A different trend was observed for the southern part of the country; a high number of cases were reported in summer and winter (Figure 2). Spring (March) findings were similar to winter, and fall (September) was similar to summer with respect to spatially reported cases. The transition from summer to winter showed a gradual shift in reported cases from south to north, whereas the transition from winter to summer showed a shift from north to south. Optimum R_p_ values for each timespan are provided in Table 1. For each season, the risk score threshold was determined using statistical parameters for 2020 and for the same evaluation of risk score threshold model performance for 2021. Overall, model performance in terms of statistical average was the same for 2021, with a slight increase or decrease in performance by season. Spring months showed a slight decrease in all three thresholds in 2021 compared with 2020. Summer (June) and fall (September) yielded similar results for 2021 compared with 2020. In summer, the model performed better for a_v_ in 2020, whereas the other two thresholds performed better in 2021 (Table 1).

**Table 1:** Optimum pixel average (R_p_) risk for predictive risk, calculated at various seasonal time steps based on given statistical parameters

Season period	Levels	Cases	R_p_	Accuracy	Precision	Sensitivity	Specificity	Average
April 2020 (Spring)	a_v_	201	0.39	92%	64%	52%	97%	76%
	a_v1_	1,558	0.5	97%	44%	59%	98%	75%
	a_v2_	2,915	0.55	98%	41%	57%	99%	74%
June 2020 (Summer)	a_v_	161	0.24	83%	46%	40%	91%	65%
	a_v1_	982	0.32	96%	36%	31%	98%	65%
	a_v2_	1,803	0.42	97%	26%	46%	98%	67%
September 3020 (Fall)	a_v_	276	0.22	82%	60%	42%	93%	69%
	a_v1_	1,204	0.28	94%	35%	34%	97%	65%
	a_v2_	2,132	0.35	98%	42%	33%	99%	68%
December 2020 (Winter)	a_v_	1,493	0.33	87%	63%	63%	92%	76%
	a_v1_	7,537	0.45	96%	50%	53%	98%	74%
	a_v2_	13,581	0.46	97%	22%	63%	97%	70%
Mar 2021 (Spring)	a_v_	412	0.39	87%	74%	34%	98%	73%
	a_v1_	1,779	0.5	95%	56%	31%	99%	70%
	a_v2_	3,146	0.55	97%	43%	28%	99%	67%
June 2021 (Summer)	a_v_	100	0.24	80%	46%	39%	89%	63%
	a_v1_	413	0.32	95%	45%	31%	98%	67%
	a_v2_	726	0.42	98%	62%	12%	100%	68%
September 2021 (Fall)	a_v_	1,015	0.22	81%	61%	46%	92%	70%
	a_v1_	3,462	0.28	94%	46%	44%	97%	70%
	a_v2_	5,908	0.35	97%	39%	25%	99%	65%

During the first 21 days of April 2020, a total of c. 603,020 COVID-19 cases were reported in the United States. New York State was the most affected, with the top five counties in the country having more than 20,000 daily reported cases. During this period, the Northeastern part of the country accounted for most of the reported cases, as shown in Figure 2. For that same period, the model predicted a higher risk score in the northeastern region than in the western and central regions (Figure 1). The dynamic component of the model (climate/weather) showed high risk score in north and central parts of the United States. However, due to spatial variability in the static component (socioeconomical), the northeastern part of the country exhibited higher risk than the western and central regions. Reported cases were at a county scale (Figure 2), whereas the predicted risk score was at a finer scale of 1 km × 1 km, with the model risk score translated to the following for validation.

### Model validation on pixel scale (R_m_).

The potential predictive intelligence at a fine spatial resolution scale was assessed by analyzing individual pixels for a particular county with the highest risk score (R_m_) for April 2020 for each reported case threshold. R_m_ risk score threshold values for a_v_, a_v1_, and a_v2_ were 0.57, 0.62, and 0.7, with average statistical values 49%, 55%, and 58%, respectively (Table 2). The model was evaluated for the four seasons of 2020 and 2021, showing comparable results in terms of risk score threshold and model performance, with a slight improvement for June 2021, compared with 2020, in terms of average statistical values (Table 2).

**Table 2:** Optimum maximum pixel (R_m_) risk for predictive risk, calculated at various seasonal time steps based on given statistical parameters

Season period	Levels	Cases	R_m_	Accuracy	Precision	Sensitivity	Specificity	Average
April 2020 (Spring)	a_v_	201	0.57	62%	14%	55%	63%	49%
	a_v1_	1,558	0.62	80%	6%	51%	81%	55%
	a_v2_	2,915	0.7	87%	5%	50%	88%	58%
June 2020 (Summer)	a_v_	161	0.35	73%	30%	56%	76%	59%
	a_v1_	982	0.5	93%	21%	41%	95%	62%
	a_v2_	1,803	0.5	94%	11%	51%	94%	62%
September 2020 (Fall)	a_v_	276	0.31	73%	40%	67%	74%	64%
	a_v1_	1,204	0.49	95%	40%	28%	98%	65%
	a_v2_	2,132	0.54	98%	41%	32%	99%	67%
December 2020 (Winter)	a_v_	1,493	0.56	83%	53%	75%	85%	74%
	a_v1_	7,537	0.57	79%	15%	92%	78%	66%
	a_v2_	13,581	0.57	76%	5%	92%	76%	62%
March 2021 (Spring)	a_v_	412	0.57	76%	40%	69%	78%	66%
	a_v1_	1,779	0.62	81%	17%	70%	82%	62%
	a_v2_	3,146	0.7	88%	12%	58%	89%	62%
June 2021 (Summer)	a_v_	100	0.35	70%	34%	63%	72%	60%
	a_v1_	413	0.5	93%	31%	36%	96%	64%
	a_v2_	726	0.5	94%	17%	42%	95%	62%
September 2021 (Fall)	a_v_	1,015	0.31	65%	37%	81%	60%	61%
	a_v1_	3,462	0.49	93%	41%	31%	97%	66%
	a_v2_	5,908	0.54	97%	47%	17%	99%	65%

Overall performance of R_p_ was better than R_m_ in validating the model at the county scale for reported disease data, as expected due to similar spatial resolution of R_p_ and disease data. However, R_m_ had high sensitivity, indicating ability to capture the reported condition, namely number of cases greater than average number of cases reported. Therefore, Rm is concluded the better tool for surveillance at the neighborhood (finer) level between the two categorized risk scores.

### Validation using wastewater sampling for SARS-CoV-2.

**Figure 3.**
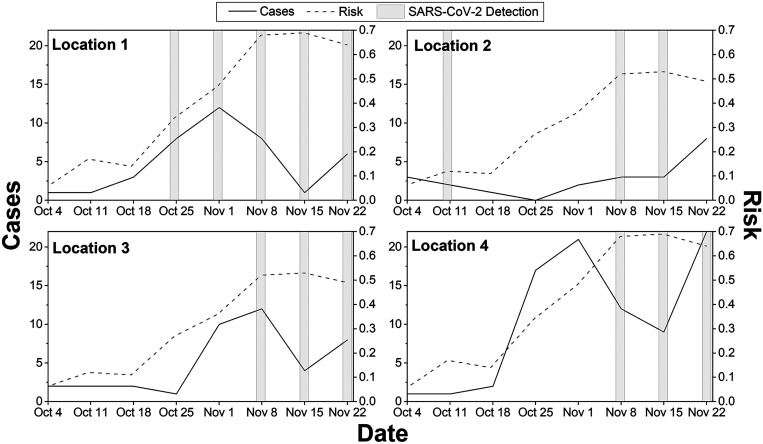
Risk scores, reported cases, and SARS-CoV-2 detection for four locations in Urbana–Champaign, IL. The dotted line represents predicted risk on that date calculated for the next 2–3 weeks. Dash-dotted line represents cases reported over the next 2 weeks (i.e., cases indicated on November 1 represents cases reported between November 1 and November 14). The bar plot represents detection of SARS-CoV-2 within the next 7 days.

**Figure 4.**
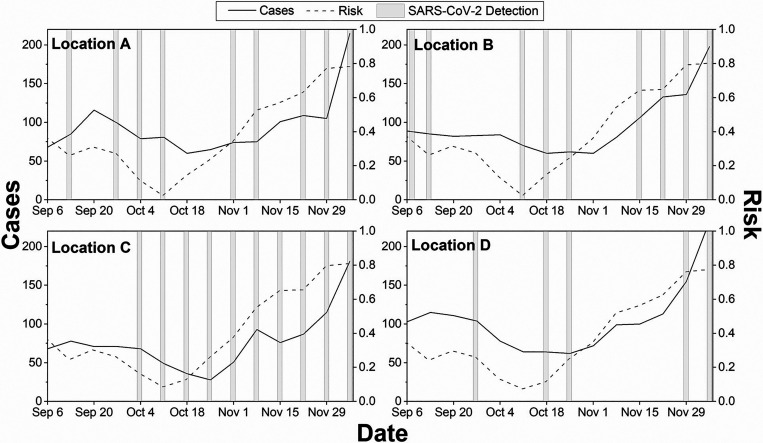
Risk scores, reported cases, and SARS-CoV-2 detection at four locations in Baltimore, MD. The dotted line represents risk, predicted on that date, calculated for next 2–3 weeks. Dash-dotted line represents the reported cases in the next 2 weeks. For example, cases on November 1 represents cases reported between November 1 and November 14. The bar represents detection of SARS-CoV-2 over the next 7 days.

Wastewater sampling was conducted in Fall 2021, September to November. With calculated accuracy at the county level, we assumed the forecasted risk score at 1-km resolution would be comparable. On the basis of these risk scores, we identified neighborhoods with high risk within Champaign, IL, and Baltimore, MD, counties. Four locations, each within the Urbana region of Champaign and the Baltimore region, were shortlisted for wastewater sampling for SARS-CoV-2, based on the predictive risk maps. In Urbana, location 1 is home to many off-campus, multiresident housing and restaurants frequently visited by students, staff, and faculty. University of Illinois required students to test for COVID-19 every 2 days. Location 2 comprises apartments and single-family housing for residents who may not work for the university but have access to free testing provided by the University of Illinois. Location 3 is a single-house family neighborhood with residents who may not work for the University of Illinois. Location 4 is also a single-house family neighborhood but includes a nearby school. In Baltimore, all four locations are in an urban setting. Figures 3 and 4 show weekly forecasted risk for fall months, wastewater monitoring results, and the human clinical test data for all eight locations. Figures indicate COVID-19 risk scores began to increase around October 18 in Urbana and October 11 in Baltimore at all locations and, with some lag, reported COVID-19 cases increased. The results show that SARS-CoV-2 was detected in wastewater samples at all locations after the risk scores predicted a potential increase, with the exception of 2 weeks at location 2 in Urbana and some locations in Baltimore.

We determined model effectiveness at neighborhood scale for the predictive risk model using Pearson correlation, as shown in Tables 3 and 4. Three categories of reported cases: 1 week, where cases reported the following week were considered; 2 weeks, where cases were reported within the next 2 weeks of calculated risk; and 3 weeks, where cases reported within the next 3 weeks of calculated risk were considered. Results showed significant association between predicted risk score and reported number of cases (0.52–0.88) for Urbana, as shown in Table 3. Location 1 exhibited the highest correlation of 0.57 for 1 week reported cases, and location 2 exhibited highest correlation of 0.79 for cases over 3 weeks. For locations 3 and 4, correlation varied between 0.57–0.88 and 0.65–0.77, respectively, being highest for locations 3 and 4 at 1 and 3 weeks, respectively. Correlation for all locations combined varied between 0.52 and 0.56, the highest within 2 weeks. In Baltimore, locations B, C, and D exhibited highest correlation, namely 0.81, 0.78, and 0.78, respectively, for 2-week cumulative cases, and location A exhibited highest correlation of 0.72 for 3-week cumulative number of reported cases, as shown in Table 4. Correlation was the highest for 2-week cumulative data in the combined series.

**Table 3:** Pearson correlation coefficient between predicted risk and 1-, 2-, and 3-week cumulative reported cases for four locations in Urbana, Illinois

Location	Correlation coefficient (*r*)/ *P*-value (*P*)	1 week	2 weeks	3 weeks
Location 1	*r*	0.57	0.41	0.28
	*P*	0.11	0.28	0.47
Location 2	*r*	0.37	0.56	**0.79**
	*P*	0.32	0.12	**0.01**
Location 3	*r*	**0.88**	**0.73**	0.57
	*P*	**0.002**	**0.03**	0.11
Location 4	*r*	**0.65**	**0.73**	**0.77**
	*P*	**0.06**	**0.03**	**0.02**
All locations taken together	*r*	**0.54**	**0.56**	**0.52**
	*P*	**0.0006**	**0.0003**	**0.0011**

Bold indicates *P* < 0.05.

**Table 4:** Pearson correlation coefficient between predicted risk and 1, 2-, and 3-week cumulative reported cases for four locations in Baltimore, MD

Location	Correlation coefficient (*r*)/ *P*-value (*P*)	1 week	2 weeks	3 weeks
Location A	*r*	0.40	**0.63**	**0.72**
	*P*	0.160	**0.015**	**0.003**
Location B	*r*	**0.68**	**0.81**	**0.71**
	*P*	**0.007**	**0.001**	**0.004**
Location C	*r*	**0.59**	**0.78**	**0.75**
	*P*	**0.025**	**0.001**	**0.002**
Location D	*r*	**0.63**	**0.78**	**0.73**
	*P*	**0.015**	**0.001**	**0.003**
All	*r*	**0.53**	**0.71**	**0.70**
	*P*	**0.00002**	**7E-10**	**1.4E-09**

Bold indicates *P* < 0.05.

In September, all locations exhibited lower COVID-19 risk scores, with the lowest on October 4 in Urbana and October 11 in Baltimore. Location 1, in agreement with the fall risk scores as shown in Table 2, SARS-CoV-2 RNA was detected every week between October 29 and November 30 (Figure 3). In agreement with the predicted risk, SARS-CoV2 RNA was detected in wastewater samples collected between November 12 and 16, with virus RNA concentrations higher than the detection limit but lower than the quantification limit. Similarly, only one and two new cases were reported during November 20 and November 27 weeks, respectively. The virus RNA was not detected until November 12, as shown in Figure 3. In Baltimore, at location C, SARS-CoV-2 RNA was detected in all samples, in agreement with predicted risk score (R_m_) of greater than 0.31 for the fall of that year. The virus RNA was detected in all Baltimore locations when the predicted risk score was greater than 0.31, as shown in Figure 4. When the predicted risk score was greater than 0.31, Urbana SARS-CoV-2 detection was 100%, 50%, 75%, and 60% for locations 1, 2, 3, and 4 and in Baltimore, detection success was 83%, 67%, 100%, and 33% at locations A, B, C, and D, respectively. In November, the risk score was higher, with a large number of cases and successful virus RNA detection in the wastewater. All locations showed an increase in the number of reported cases during the week of December 4.

## DISCUSSION

Progression of COVID-19 is traditionally monitored by counting the number of reported cases of individuals testing positive for the presence of SARS-CoV-2, which is generally prompted by presentation of symptoms. However, presymptomatic and/or asymptomatic COVID-19 transmission has been observed.^49^ Clinical testing programs have been useful in professional settings, but selective testing mandates only apply to certain groups or regions, thus providing limited information only on a subset of individuals compared with the total population. Similarly, self-tests have become increasingly popular but rely on individuals to perform tests properly and report positive results to health departments. Hence, case reports for estimations of COVID-19 progression have the potential to be biased. As a complement to clinical testing, WS has been used for early detection of community-wide disease prevalence, including COVID-19^50^ because the wastewater microbiome provides useful public health information to proactively detect and characterize pathogenic agents circulating in a community.^13^ However, it should be noted that WS requires assessment of chemically and biologically complex samples that have the potential to be impacted by weather/climatic processes, such as rainfall, and other inhibitory compounds, such as industrial waste, that can interfere with accurate measurements. Another caveat is that there is currently no standardized procedure for WS sample collection/processing methodologies, which can introduce detection and/or quantification bias. Hence, in monitoring a given community, WS is most useful in municipalities with sewer collection systems, and the representation of rural towns may be diminished. Nonetheless, WS has been shown to be a valuable public health tool to study the emergence and spread of COVID-19 proactively.

Emerging infectious respiratory diseases like COVID-19 cannot be predicted with great accuracy, mainly because the virus is novel and its pathways of transmission in the human population remain to be described in detail. Therefore, we argue that decision-making encompassing a prediction intelligence system should be devised rather than an approach for an absolute prediction. A complex climate–sociological hypothesis for the spread of COVID-19 in the human population was reported in our previous study.^17^ It indicated that the virus exhibits seasonality during cold and warm environments and is a function of seasonal change in human behavior. On the basis of this hypothesis, a prediction architecture was constructed (pixel and county scale) and validated for four seasonal periods in the continental United States. A key conclusion is that model validation was achieved on a seasonal basis, with indication that the disease may be becoming endemic. The lack of satisfactory statistical metrics on an annual scale further corroborates the finding that the virus may now be circulating in the human population. Outbreaks appear to be a function of cold and warm regions, depending on time and geographic space. Second, the performance of the model was better when computed risk scores were averaged over the county (R_p_) compare to maximum computed risk score (R_m_). However, from the standpoint of decision-making, both R_p_ and R_m_ are likely to lead to different intervention strategies. For example, selection of at-risk counties should be made based on R_p_; in contrast, R_m_ will dictate the location for wastewater sampling (i.e., microbiological identification for presence of the virus). Third, lead time is calculated using the model, and was validated using wastewater sampling, hence emphasizing the importance of microbiological monitoring of sewer systems for public health. A nationwide wastewater sampling grid or nodes should be established to monitor the presence and movement of microbial pathogens within human populations as a significant public health tool.

## CONCLUSION

Results of the study reported here are promising: COVID-19 can be managed with systematic information from the model, combined with microbiological sampling of wastewater. Although vaccinations lower the chance that individuals suffer serious disease or hospitalization, the likelihood of infection in the human population remains a constant threat. Innovative mitigation and intervention strategies that integrate interdisciplinary knowledge (i.e., microbiology, sociology, climate, earth sciences, and public health, all at the global scale) will be required for future pandemics.

## Supplemental Materials

10.4269/ajtmh.23-0077 Supplemental Materials
