# Supplementary material for: Building Environmental and Sociological Predictive Intelligence to Understand the Seasonal Threat of SARS-CoV-2 in Human Populations
Source: Am J Trop Med Hyg. 2024 Feb 6;110(3):518–28. doi: 10.4269/ajtmh.23-0077 (PMC10919182; doi:10.4269/ajtmh.23-0077)
Supplement: Supplemental Materials [file tpmd230077.SD1.pdf]

Building Environmental and Sociological Predictive Intelligence to Understand the Seasonal Threat of SARS-CoV-2 in Human Populations

Moiz Usmani[1], Kyle Brumfield[2,3], Bailey Magers[1], Aijia Zhou[4], Chamteut Oh[4], Yuqing Mao[4], Willam Brown[4], Arthur Schmidt[4], Chang-Yu Wu[5], Joanna L Shisler[6], Thanh H. Nguyen[4], Anwar Huq[2,3], Rita Colwell[2,3], and Antarpreet Jutla[1#]

[1]GeoHealth and Hydrology Laboratory, Department of Environmental Engineering Sciences, University of Florida, Gainesville, FL, USA.

[2]Maryland Pathogen Research Institute, University of Maryland, College Park, MD 20742, USA.

[3]University of Maryland Institute for Advanced Computer Studies, University of Maryland, College Park, MD 20742, USA

[4]Department of Civil and Environmental Engineering, University of Illinois at Urbana-Champaign, Urbana, IL, USA

[5]Department of Environmental Engineering Sciences, University of Florida, Gainesville, FL, USA.

[6]Department of Microbiology, University of Illinois at Urbana-Champaign, Urbana, IL, USA,

# Corresponding author: ajutla@ufl.edu

Supplementary Section

Supplementary Figures

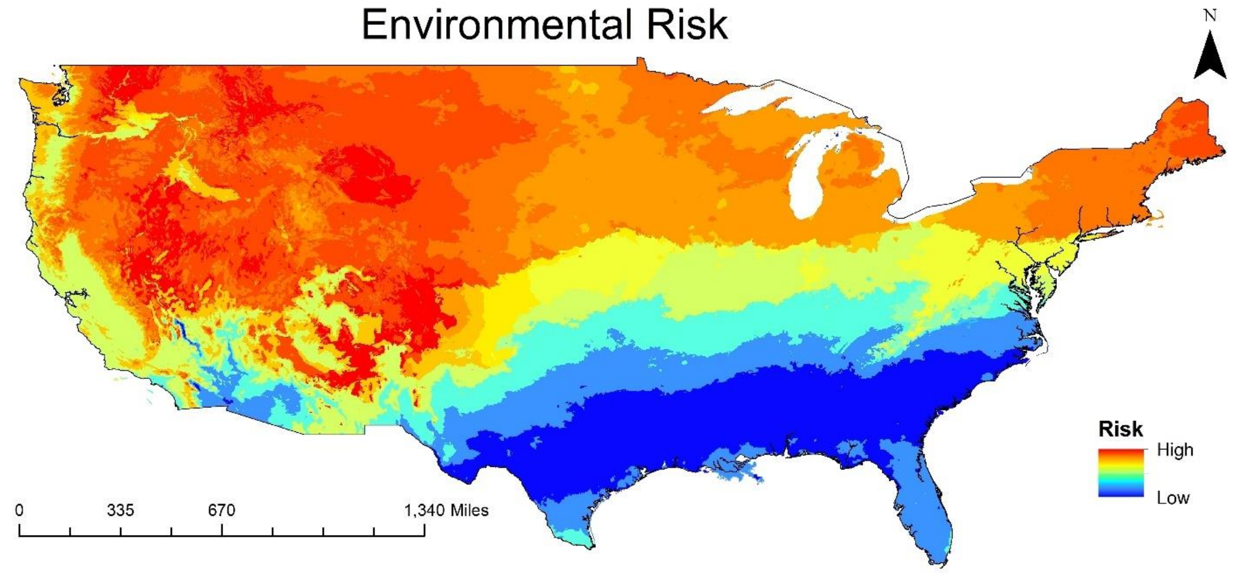

Figure S1: Environmental risk: first component of COVID-19 risk, calculated using only climate variables.

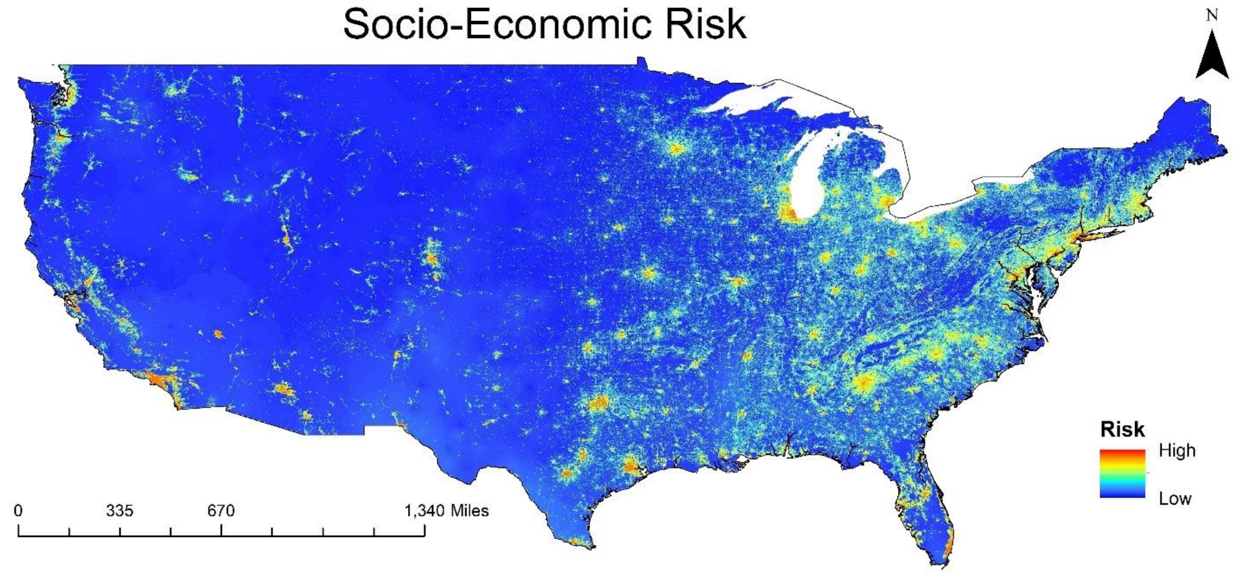

Figure S2: Socio-economical risk: second component of COVID-19 risk, calculated using socio-economic variables.

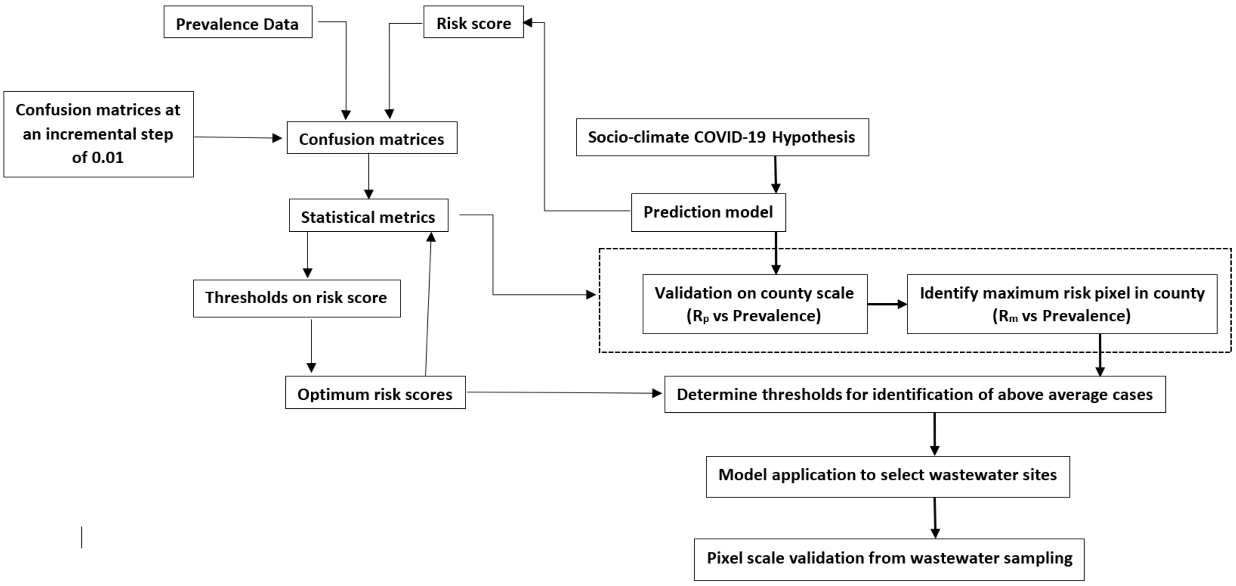

Figure S3: Conceptual structure of the study presented through a flowchart.

Supplementary Table

Table S1: Environmental component of the risk model.

Model<br>Air temperature and difference between temperature and D_t	Accuracy	Precision	Sensitivity	Specificity	Average
0.9 and 0.1	92%	74%	37%	99%	76%
**0.8 and 0.2**	**93%**	**75%**	**40%**	**99%**	**76%**
0.7 and 0.3	89%	41%	12%	98%	60%
0.6 and 0.4	89%	37%	15%	97%	59%
0.5 and 0.5	90%	41%	10%	98%	60%

Weights of environmental parameters; temperature and difference between temperature and D_t varied between 0.5 to 0.9 and 0.1 to 0.5, respectively. The model with summation of parameters at 0.8 and 0.2 exhibited the best results.

Table S2: Co-ordinates of sampling locations**.**

Sampling sites	Latitude	Longitude
**Location A**	39.22975	-76.6018
**Location B**	39.26403	-76.6408
**Location C**	39.31655	-76.6318
**Location D**	39.2784	-76.5409
**Location 1**	40.10905	-88.232
**Location 2**	40.13096	-88.2627
**Location 3**	40.09822	-88.28
**Location 4**	40.10589	-88.1726

Table S3. RT-qPCR primer information.

Target species	Target gene or	Primer name	Sequence (5'-3')	GC content	Tm (°C)	Amplicon size (bp)

	mutation			(%)		
SARS-CoV-2	N[1)]	CDC_N1_Forward	GACCCCAAAATCAGCGAAAT	45.0	61.1	72
		CDC_N1_Reverse	TCTGGTTACTGCCAGTTGAATCTG	45.8	64.5	
		CDC_N1_Probe	ACCCCGCATTACGTTTGGTGGACC	58.3	70.3	

Table S4: Confusion matrix for April 1 – April 21 to determine Rp.

Risk	Tp M	Tp 1S	Tp 2S	Fn M	Fn 1S	Fn 2S	Tn M	Tn 1S	Tn 2S	Fp M	Fp 1S	Fp2S
0.2	294	67	42	12	1	0	139	150	151	2618	2845	2870
0.21	292	67	42	14	1	0	235	248	249	2522	2747	2772
0.22	288	66	41	18	2	1	384	400	401	2373	2595	2620
0.23	284	65	40	22	3	2	557	576	577	2200	2419	2444
0.24	279	65	40	27	3	2	741	765	766	2016	2230	2255
0.25	274	65	40	32	3	2	947	976	977	1810	2019	2044
0.26	269	63	40	37	5	2	1175	1207	1210	1582	1788	1811
0.27	264	63	40	42	5	2	1390	1427	1430	1367	1568	1591
0.28	258	61	39	48	7	3	1595	1636	1640	1162	1359	1381
0.29	248	61	39	58	7	3	1802	1853	1857	955	1142	1164
0.3	239	61	39	67	7	3	1972	2032	2036	785	963	985
0.31	233	61	39	73	7	3	2119	2185	2189	638	810	832
0.32	223	60	39	83	8	3	2245	2320	2325	512	675	696
0.33	214	59	39	92	9	3	2350	2433	2439	407	562	582
0.34	206	59	39	100	9	3	2429	2520	2526	328	475	495
0.35	197	58	38	109	10	4	2502	2601	2607	255	394	414
0.36	188	57	37	118	11	5	2545	2652	2658	212	343	363
0.37	177	56	37	129	12	5	2591	2708	2715	166	287	306
0.38	168	55	36	138	13	6	2632	2757	2764	125	238	257
0.39	159	55	36	147	13	6	2652	2786	2793	105	209	228
0.4	145	55	36	161	13	6	2670	2818	2825	87	177	196
0.41	132	52	36	174	16	6	2690	2848	2858	67	147	163
0.42	129	52	36	177	16	6	2703	2864	2874	54	131	147
0.43	121	52	36	185	16	6	2717	2886	2896	40	109	125
0.44	106	50	34	200	18	8	2721	2903	2913	36	92	108
0.45	97	48	34	209	20	8	2724	2913	2925	33	82	96
0.46	88	46	33	218	22	9	2725	2921	2934	32	74	87
0.47	83	43	33	223	25	9	2727	2925	2941	30	70	80

0.48	80	43	33	226	25	9	2728	2929	2945	29	66	76
0.49	74	41	32	232	27	10	2730	2935	2952	27	60	69
0.5	67	40	32	239	28	10	2730	2941	2959	27	54	62
0.51	62	37	30	244	31	12	2736	2949	2968	21	46	53
0.52	59	35	30	247	33	12	2738	2952	2973	19	43	48
0.53	53	33	28	253	35	14	2739	2957	2978	18	38	43
0.54	49	30	25	257	38	17	2741	2960	2981	16	35	40
0.55	44	29	24	262	39	18	2741	2964	2985	16	31	36
0.56	38	24	20	268	44	22	2742	2966	2988	15	29	33
0.57	33	20	16	273	48	26	2742	2967	2989	15	28	32
0.58	31	19	16	275	49	26	2744	2970	2993	13	25	28
0.59	30	19	16	276	49	26	2747	2974	2997	10	21	24
0.6	27	18	15	279	50	27	2748	2977	3000	9	18	21
0.61	26	17	14	280	51	28	2748	2977	3000	9	18	21
0.62	23	16	14	283	52	28	2750	2981	3005	7	14	16
0.63	22	15	13	284	53	29	2751	2982	3006	6	13	15
0.64	21	14	12	285	54	30	2751	2982	3006	6	13	15
0.65	20	14	12	286	54	30	2752	2984	3008	5	11	13
0.66	19	13	12	287	55	30	2752	2984	3009	5	11	12
0.67	18	13	12	288	55	30	2752	2985	3010	5	10	11
0.68	18	13	12	288	55	30	2752	2985	3010	5	10	11
0.69	17	12	11	289	56	31	2752	2985	3010	5	10	11
0.7	17	12	11	289	56	31	2753	2986	3011	4	9	10
0.71	14	11	10	292	57	32	2753	2988	3013	4	7	8
0.72	13	10	9	293	58	33	2753	2988	3013	4	7	8
0.73	11	9	8	295	59	34	2753	2989	3014	4	6	7
0.74	10	8	7	296	60	35	2753	2989	3014	4	6	7
0.75	10	8	7	296	60	35	2753	2989	3014	4	6	7
0.76	9	8	7	297	60	35	2754	2991	3016	3	4	5
0.77	9	8	7	297	60	35	2754	2991	3016	3	4	5
0.78	7	6	6	299	62	36	2754	2991	3017	3	4	4
0.79	7	6	6	299	62	36	2754	2991	3017	3	4	4
0.8	7	6	6	299	62	36	2756	2993	3019	1	2	2
0.81	7	6	6	299	62	36	2757	2994	3020	0	1	1
0.82	5	5	5	301	63	37	2757	2995	3021	0	0	0
0.83	5	5	5	301	63	37	2757	2995	3021	0	0	0
0.84	2	2	2	304	66	40	2757	2995	3021	0	0	0
0.85	2	2	2	304	66	40	2757	2995	3021	0	0	0
0.86	1	1	1	305	67	41	2757	2995	3021	0	0	0
0.87	0	0	0	306	68	42	2757	2995	3021	0	0	0
0.88	0	0	0	306	68	42	2757	2995	3021	0	0	0
0.89	0	0	0	306	68	42	2757	2995	3021	0	0	0
0.9	0	0	0	306	68	42	2757	2995	3021	0	0	0

0.91	0	0	0	306	68	42	2757	2995	3021	0	0	0
0.92	0	0	0	306	68	42	2757	2995	3021	0	0	0
0.93	0	0	0	306	68	42	2757	2995	3021	0	0	0
0.94	0	0	0	306	68	42	2757	2995	3021	0	0	0
0.95	0	0	0	306	68	42	2757	2995	3021	0	0	0
0.96	0	0	0	306	68	42	2757	2995	3021	0	0	0
0.97	0	0	0	306	68	42	2757	2995	3021	0	0	0
0.98	0	0	0	306	68	42	2757	2995	3021	0	0	0
0.99	0	0	0	306	68	42	2757	2995	3021	0	0	0
1	0	0	0	306	68	42	2757	2995	3021	0	0	0
